# Democratising health and social care research through long-term public involvement and engagement: a qualitative process evaluation of the Community Research and Engagement Network (CoREN)

**DOI:** 10.1186/s40900-026-00897-2

**Published:** 2026-05-23

**Authors:** Steven Dodd, Koser Khan, Bethany Gill, Katerina Panagaki, Alison Doherty, Hilary Garrett, Jane Cloke

**Affiliations:** 1 Division of Health Research, Faculty of Health & Medicine, Lancaster University, Bailrigg, Lancaster, LA1 4YG UK; 2 Synthesis, Economic Evaluation and Decision Science (SEEDS) Group, Applied Health Research Hub, University of Lancashire, Preston, PR1 2HE UK; 3 National Institute for Health and Care Research Applied Research Collaboration North West Coast (NIHR ARC NWC), Liverpool, UK; 4 Institute of Population Health, University of Liverpool, Liverpool, UK

**Keywords:** Community engagement, Co-production, Deliberative democracy, Health and social care research, Health inequalities, Knowledge mobilisation, Participatory research, Public involvement, Qualitative evaluation

## Abstract

**Background:**

Interest in public involvement in UK health and social care research continues to grow, yet many initiatives are short-lived and offer public collaborators little meaningful influence. Established in 2019 within the NIHR Applied Research Collaboration North West Coast (ARC NWC), the Community Research and Engagement Network (CoREN) was created to provide a sustained platform for co-production of health and social care research. This article reports a theory-informed qualitative process evaluation of the CoREN, examining its progress towards democratic, long-term co-production and the factors that enable or impede it.

**Methods:**

Methodologically, a qualitative, relational approach informed by deliberative democratic theory was used. Data were collected via semi-structured interviews with CoREN Leadership Group members (n=6) and affiliated researchers (n=4) and three focus groups with community members and partners (total n=17). Framework analysis was applied using a priori and inductive codes.

**Results:**

Participants described the CoREN as a coordinating nexus that connects researchers and community organisations, brokers relationships and “plants seeds” that later grow into co-produced projects. Other key impacts included amplifying community voices in the research process, helping to equalise power dynamics in researcher-community relationships, and building research literacy and capacity within communities. Challenges included a lack of clarity regarding the CoREN’s role and structure, bureaucratic inertia, and difficulties in including a representative range of diverse and less privileged communities across the region.

**Conclusions:**

The CoREN model demonstrates the promise of sustained, non-project-bound engagement networks as a means of strengthening democratic participation in research and building capacity for future co-production. Sustained investment, ongoing feedback and evaluation, and a commitment to overcoming structural barriers to participation are needed to ensure that progress towards equitable community-led research is maintained.

**Supplementary Information:**

The online version contains supplementary material available at 10.1186/s40900-026-00897-2.

## Abstract (plain language summary)

This article reports the results of an evaluation of the Community Research and Engagement Network (CoREN), an initiative started in 2019 in the North West of England, aiming to give local people, voluntary and community organisations, practitioners and researchers a longer-term way to work together on health and social care research. To understand the CoREN’s impacts and how it works in practice, we spoke to six members of its Leadership Group, four academic researchers who had worked with the CoREN, and held three focus groups with community members with experience of the CoREN. Participants told us the CoREN helps to link up partners who would not otherwise meet, bridging the gap between academic work and real-world problems, and “planting seeds” that later grow into joint research projects. Other key impacts included boosting the voices of communities in research, helping to balance power in research teams between academics and members of the public, and increasing community understanding of research. However, the network also faces challenges. Some participants were confused about its exact role and felt working with it could be slow or formal. Although it has made progress in including a range of diverse communities in the region, there remains room for improvement. Overall, the CoREN shows how a long-term network can support greater patient and public involvement in research. Further investment, clearer communication about its role, and stronger communication with under-represented groups would help it maintain progress and reach its potential.

**Supplementary Information:**

The online version contains supplementary material available at 10.1186/s40900-026-00897-2.

## Background

In the UK, public involvement plays an increasingly prominent role in research, with the National Institute for Health and Care Research (NIHR) positioning it as a core principle of its work to ensure research “reflects what matters to people and communities, and meets their needs” [1]. Co-production is seen to take participation a level further, acting as a more collaborative and intensive form of involvement, based on more equal and sustained partnerships between researchers and the public, including shared power and joint decision-making throughout the research cycle [2, 3]. Interest in these ideals has come about in a time of explosive growth in participatory initiatives, alongside an ever-widening range of vocabularies and concepts used to describe them [4–8]. Navigating this increasingly nebulous space, and applying some conceptual precision using relevant theory, this article presents a theoretically informed qualitative process evaluation of the Community Research and Engagement Network (CoREN), and its progress towards supporting sustained, democratic co-production of health and social care research^1^.

### Introduction to the CoREN

The CoREN’s origins lie in the NIHR Collaboration for Leadership in Applied Health Research and Care (CLAHRC) North West Coast’s Neighbourhood Resilience Programme (2016–2019), which used systems-level dialogue in nine disadvantaged neighbourhoods to identify priorities and support action on the social determinants of health. At this earlier stage, nine separate CoRENs operated as neighbourhood-based networks, each led by a local third-sector organisation that employed a facilitator to recruit and support resident advisers, who helped to deliver the resilience initiatives and build shared understanding of them.

Work by the NIHR CLAHRC highlighted the need for greater integration of the Voluntary, Community, Faith and Social Enterprise (VCFSE) sector and stronger community influence over research. With the evolution of the CLAHRC NWC into the Applied Research Collaboration North West Coast (ARC NWC) in 2019, the CoREN was reimagined as a means to help meet this need and embed more participatory ways of working across ARC NWC’s research collaborations, providing enduring institutional capacity for community involvement in health and social care research. ARC NWC is one of several regional partnerships funded by the National Institute for Health and Care Research (NIHR) to carry out applied research that brings together universities, the NHS, local authorities and others. Covering a large and socioeconomically diverse area from South Cumbria to Cheshire, the region includes some of the most deprived neighbourhoods in England, where many communities face persistent health inequalities driven by the intersecting effects of social, economic and cultural marginalisation.

**Fig. 1:**
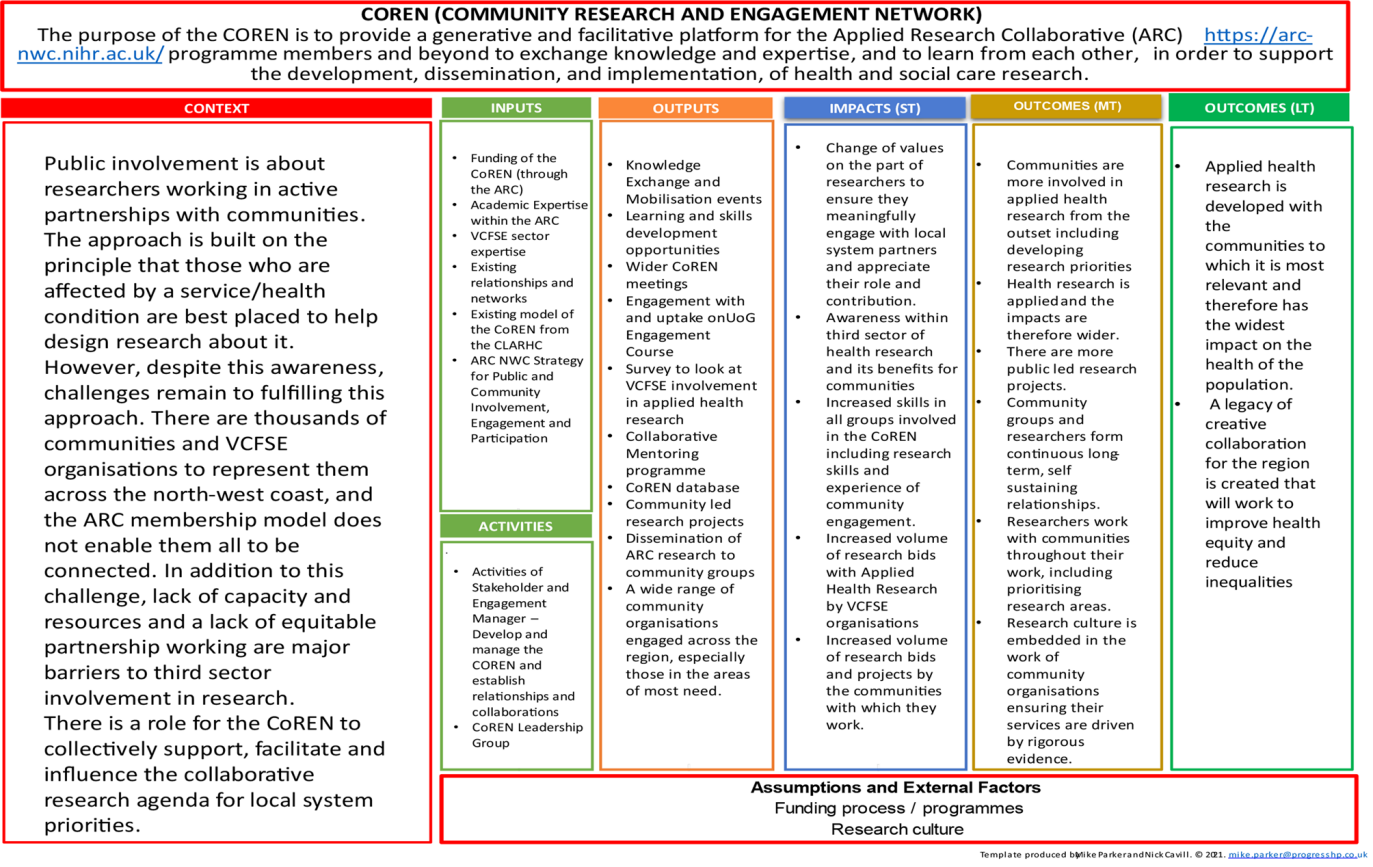
Community Research and Engagement Network logic model: context, inputs, activities, outputs, short-term impacts, medium-term outcomes, and long-term outcomes

As set out in its logic model (see Fig. 1), the CoREN’s overarching goal is “to provide a generative and facilitative platform for ARC [NWC] members^2^ and beyond to exchange knowledge and expertise, and to learn from each other, in order to support the development, dissemination and implementation of health and social care research”. In service of this goal, the CoREN enables members of numerous communities and VCFSE organisations to contribute their locally held knowledge to identifying and tackling public health challenges, shifting agenda-setting power toward communities experiencing poorer outcomes so that research addresses the conditions that drive health inequalities. As a long-term and sustained network, rather than a series of time-limited individual research projects, the CoREN aims to forge enduring relationships between groups and individuals who may otherwise work separately from one another, allowing often-overlooked communities to exert ongoing influence.

The CoREN is overseen by a nine-member Leadership Group, within which strategic direction and priorities are agreed through discussion. At inception, the membership^3^ of the Leadership Group was drawn from VCFSE organisations with whom ARC NWC (and its predecessor, CLAHRC NWC) already had established working relationships. As the CoREN developed, additional organisations were invited to strengthen representation across the ARC NWC region, particularly geographically. Later, two further organisations joined via the local Research Engagement Network (REN) programme, an NHS England-funded initiative supporting VCFSE-led work to involve communities that are often excluded from research.

The Leadership Group’s remit and ways of working developed through ongoing meetings and partnership discussions as the network grew. Members are expected to attend Leadership Group meetings, contribute to the CoREN’s strategic direction, host or support at least one CoREN engagement activity per year, and share CoREN updates and opportunities through their organisational networks. Members’ roles often extend beyond these core expectations into brokering and relationship-building, particularly through connecting community organisations with researchers.

To deliver agreed objectives and tasks, the work of the network is supported by a full-time Stakeholder and Engagement Manager (SEM), employed by and based within ARC NWC. The role of the SEM is to provide input to strategic discussions led by the leadership group, and to manage day-to-day coordination while supporting delivery of CoREN activities. The SEM also brokers one-to-one links and mentoring partnerships, matching researchers with community organisations whose lived experience or professional expertise can shape research. Delivery was guided by an action plan co-developed by the SEM and the Leadership Group and agreed with the ARC NWC management team. Leadership of the ARC NWC had oversight of the CoREN through regular updates and reporting from the SEM, responding with oversight, feedback, and support for new ideas and ways of working. Both the Leadership Group roles and the SEM post are funded by the ARC NWC. Around this core governance structure sits an open membership^4^ of community groups, NHS and local-authority practitioners, academics, and interested residents. Members are contacted through a mailing list and events designed to bring members of the public, practitioners and academics together to identify shared priorities, align research with community needs, and plan and conduct research projects collaboratively.

The CoREN’s core activities have included a Collaborative Mentoring programme pairing researchers with VCFSE partners in reciprocal roles; a knowledge-mobilisation webinar series on priority topics; periodic Collaboration Cafés that mix short talks with table discussions to seed projects; day-to-day brokering and signposting between researchers and VCFSE groups; short, practical training and support, guided by a region-wide 2022 survey of VCFSE organisations and community groups on needs; co-developing the Community Ready Researchers short online course with an external academic partner; and supporting NHS England engagement funding applications. Taken as a whole, these activities contribute to the network’s long-term aspiration of contributing to a research ecosystem in which communities set priorities, co-design research, and remain actively involved throughout the research cycle into implementation.

#### The CoREN’s long-term, non-project-bound model

Though its long-term approach is rare, the CoREN model of collaboration is part of a small but growing tradition in health and social care research^5^. The longer-term commitment of resources and sustained model of engagement allow relationships and trust to deepen and mean it is not forced to create a new partnership for every grant, or to close collaborations down when funding ends^6^. As a result, it doesn’t become trapped in the stop-start cycle of “projectisation” [9] that can restrict sustained dialogue [2]. By giving it some distance from the “grants culture,” it avoids reducing patient and public involvement to a temporary box-ticking requirement for securing funding [2].

Given its departure from more “projectified” approaches to public involvement, the CoREN may be better positioned to contribute to research about the underlying power structures that time-limited projects tend to treat as outside their funding remit [10]. With its stable, long-term group of collaborators, it is also more likely to be able to build the trust and shared language and insight [10] needed to challenge structural inequities in society and research.

## Methods

### Methodological approach

We adopt a qualitative, relational approach to this evaluation, informed by deliberative democratic theory [14, 18]. This is partly in response to critiques that metric-led assessments of participatory research tend to focus on short-term, quantifiable outcomes [10, 11], potentially overlooking the subtle, harder-to-measure relational changes the CoREN aspires to create, and the fluid and unpredictable nature of its impacts [12, 13].

Our approach is influenced by Greenhalgh et al. [14] who suggest that metric-led approaches rely on linear assumptions of input and output, neglecting the more fluid, contested, and emergent nature of co-production, where causal links are diffuse and outcomes unfold over extended periods. Instead, more can be gained by studying relational questions of how knowledge is generated, who participates, and how power is shared [14].

#### A relational approach to evaluation

The relational approach to evaluation we endorse [14–17] treats impact as the formation of new or reconfigured relationships, affecting who can act and decide, with effects that can ripple out across the wider social network. Such approaches track subtle changes such as shared language and joint decision-making that create capacity for more fundamental relational changes in the future. This aligns with the CoREN’s aim to seed future co-production by reconfiguring local research relations. As such, we use a relational lens to focus on the quality of cross-sector relationships as both outcomes of the network and mechanisms through which later, downstream impacts may occur.

#### Deliberative theoretical lens

Deliberative democratic theory [18] provides a complementary lens, focusing on the quality of discussion [18, 19], and whether the CoREN’s activities provide sufficient opportunity for inclusive participation. It judges legitimacy by the quality of interactions such as reason-giving, mutual listening, testing claims, and fair inclusion in who is heard [18, 19]. This echoes the CoREN logic model’s democratic aims, its use of deliberative language, its commitment to sustained dialogue, and the CoREN’s creation of spaces for deliberation. In practice, we draw on deliberative-democratic ideas to examine whether the CoREN creates conducive conditions for collaborative work, including opportunities for reason-giving, mutual listening, and the ability to challenge claims made by others.

Contemporary deliberative theory and relational evaluations of co-production are complementary because they share the view that legitimacy depends on how participants interact. Deliberative scholars maintain that authority emerges through reason-giving, mutual listening and testing of competing claims, while, similarly, relational evaluators of patient and public involvement show how status is negotiated [20], knowledge is exchanged and preferences are reshaped during the research process [21].

In the sections that follow, we examine the CoREN’s development and outcomes through this combined lens. While the empirical findings are predominantly presented in practical terms, the deliberative and relational theories outlined above remain the backdrop to our analysis and serve as a basis for discussing the extent of the CoREN’s successes.

### Methods and analysis

#### Research objectives

- Explore the role of the CoREN in practice, including how it supports co-production.
- Examine how the CoREN shapes relationships and influence among community members, practitioners and researchers.
- Assess the CoREN’s progress in promoting public involvement in research, including facilitators, barriers, and challenges.
- Identify impacts and tensions and provide recommendations for improvement.

Qualitative interviews and focus groups were conducted between November 2023 and March 2024. All members of the ARC NWC CoREN Leadership Group were invited to take part in a semi-structured interview (*n* = 9) and six participated. Approximately 20 ARC NWC researchers were also invited, identified via the CoREN mailing list and through direct contacts with research staff known to have engaged with the CoREN. Researcher uptake was lower than anticipated (*n* = 4 participated). We recorded no explicit refusals and no withdrawals after consent; non-participation mainly reflected non-response and limited availability.

Focus group participants were drawn from the CoREN database, which includes individuals who have engaged with the CoREN. The sample for the three focus groups comprised 17 participants, each of whom received a £20 shopping voucher as compensation for their participation. Invitations were emailed to the CoREN contact database (just over 200 contacts at the time) and were also circulated by Leadership Group organisations through their own networks and mailing lists. Because invitations were cascaded in this way, we cannot estimate how many people ultimately received the invitation and therefore cannot state an overall response rate. No repeat interviews or focus groups were conducted and all eligible individuals who expressed interest were included.

Interviews with academic researchers were conducted by SD (male) and interviews with CoREN Leadership Group members by KK (female). Focus groups were facilitated by BG (female), JC (female) and AD (female). All researchers have formal training in qualitative methods and several years’ experience of interviews and/or focus group facilitation. At the time of the study, all facilitators held university posts funded by ARC NWC, and some had prior professional contact with participants through ARC NWC/CoREN activities. To reduce any perceived pressure to be positive, we used neutral study materials, reiterated confidentiality and independence at the outset, and ensured interviewers had no line-management relationship with participants. Participants were informed that the researchers were conducting an evaluation on behalf of ARC NWC to understand and improve the CoREN’s role and progress.

Participant demographic details and organisational identifiers were not recorded because the core of the network is small and disclosing even limited background information could compromise participants’ confidentiality.

Semi-structured topic guides (Supplementary File 1) explored current research practice and culture (pre/post CoREN), perceived benefits and challenges of engaging with the CoREN, facilitators and barriers to co-production, how the CoREN contributes to co-production, influences on research and community-engagement skills, motivations and confidence for participating in research, and recommendations for future working in the CoREN. Guides were reviewed within the team and piloted informally with one colleague. All interview and focus group participants received an information sheet and provided consent before participation. Data collection was conducted via Microsoft Teams, with recordings and transcriptions completed using its built-in features.

Interviews lasted between 45 and 60 min, and focus groups lasted approximately 90 min. Field notes were kept after each session and used to inform coding. We observed diminishing novelty in codes towards the end of data collection across the combined dataset. However, given the modest number of researcher interviews, we do not claim saturation for the researcher subgroup.

Transcripts were analysed using framework analysis [22] in NVivo 12, with two researchers (SD and KK) applying a priori codes based on the topic guide and evaluation objectives. Framework analysis was preferred because it enabled us to combine a priori codes with emergent themes, meaning quotations could be linked to evaluation objectives while allowing for inductive themes to emerge.

Deliberative democratic theory and relational evaluation informed parts of our analytic focus and coding. These themes were reflected in the topic guides, which asked about control and ownership of the CoREN, as well as relational features, including trust and reciprocity, changes in confidence, motivations and research literacy, perceived shifts in research culture or attitudes, and changes in relationships, and connecting, coordinating and networking across community organisations and researchers. Of the set of a priori codes we began with, some were also derived from deliberative concerns, including levels of influence, agenda setting and ownership, whose knowledge is treated as legitimate, how inclusive participation feels in practice, and whether participants described reason-giving, mutual listening, and respectful disagreement. As our data capture participants’ accounts rather than direct observation of the CoREN’s activities, we inferred deliberative dynamics from how interactions were described.

Codes were refined iteratively, with coding consistency checked through regular review sessions between the two researchers. Transcripts were not returned to participants for comment; however, a summary of key findings was shared with the CoREN Leadership Group, and their feedback was considered when refining interpretations.

Below, we present major themes with illustrative quotations (labelled by data source) and note variant or negative cases where relevant. A list of the final analysis codes (coding framework) is provided in Supplementary File 2. Patient and public involvement in the study is reported in line with the GRIPP2 Short Form [23], and a completed checklist is provided in Supplementary File 3.

Ethics approval was granted by Lancaster University in September 2023.

## Findings

Findings are presented in a narrative below that explores participants’ understandings of the CoREN’s impacts, the factors driving its successes, the challenges it faces, and changes they propose.

### Impacts of the CoREN

#### The CoREN as a coordinating nexus for collaboration

Participants’ responses showed how the CoREN’s role was often conceptualised as a conduit for collaboration between researchers and community members, connecting community organisations and researchers to each other:

*The CoREN is about bringing together that diverse network and feeding it into a wider understanding of what research priorities might be. But also making sure that people are connected so that intelligence and knowledge are shared and used appropriately. (Researcher interview 1)*

*The CoREN is bringing those communities together with grassroots community groups*,* and to be open to the idea of engaging researchers. (Leadership Group interview 2)*

Due to its intermediary role as a nexus or bridge promoting collaboration, impacts of the CoREN highlighted by participants were often concerned with facilitating connections between disparate individuals and organisations. Part of this is the CoREN’s institutional knowledge of VCFSE organisations in the region enabling it to act as a knowledge broker, signposting and connecting diverse individuals and organisations, bridging the gaps that typically exist between them:

*Just as a resource in itself [The CoREN] is really valuable with the knowledge and expertise of those that are leading it… they’re very immediately sort of tapped into so many different networks and organisations and collaborations and very knowledgeable. (Researcher interview 2)*

Another key driver of the CoREN’s impacts through its intermediary role is the events and activities it holds to bring together a broad range of actors, including researchers, community representatives and practitioners, thereby “connecting the dots between academics, communities and industry” (Researcher interview 1) and facilitating collective reflection on population health priorities and shared research agendas.

A less readily tangible but perhaps more profound and distinctive form of impact flowing from the CoREN’s intermediary and enabling role is its creation of fertile conditions from which future research partnerships can emerge. One participant described this as planting seeds that later grow into co-produced work:

*It’s a ripple*,* isn’t it?… the impact is quite subtle at the moment. I think future impacts around coordinating and supporting big developing ideas*,* that’s a more tangible impact*,* but I think CoREN’s more subtle than that. I don’t think that’s its job to do that. I think it’s you plant some seeds and watch the tree grow. (Researcher interview 1)*

#### Bolstering community involvement in research by equalising community-researcher relationships

Closely related to its successes in acting as an intermediary nexus, participants believed the CoREN was making progress in amplifying the voice of communities in research and, in doing so, may be helping to narrow the gap between co-production ideals and day-to-day practice:

*It was good to see that community perspective getting in*,* listening to it and it being included within different projects*,* which I think is really important*,* because from my experience*,* I find that often what is in theory when it’s put into practice*,* there’s a gap. (Focus group 3 - Participant 1)*

The conduit role of the CoREN went beyond bringing researchers, members of the public and VCFSE organisations into the same spaces. It was also seen to include connecting the ARC NWC’s research priorities with community voices during research development:

*It’s [the CoREN] the mechanism by which the ARC ensures the projects we’re working on and pushing forward are actually grounded in issues experienced by member organisations and*,* more importantly*,* by individual voices. (Researcher interview 3)*

Another example of the CoREN’s impacts in equalising power relationships and increasing inclusion is the success of the Collaborative Mentoring initiative in which both participants, one primarily from a research background and the other primarily from a community background, reciprocally share knowledge and alternate roles as mentor and mentee. Predicated on the assumption of parity, and that both parties possess valuable insight, participants reported two-way learning and benefits for both parties to the initiative:

*I thought that [Collaborative Mentoring] was a really good experience because it gave us a chance to do some mentoring with the researchers*,* but it also gave a chance for the researchers to understand how much work actually goes into the frontline… And then from my perspective… it gave me a better understanding of what goes into research. (Focus group 3 - Participant 1)*

An additional impact related to equalisation of power relationships and greater inclusion is making researchers more aware of the lives of seldom-heard communities and those with marginalised identities who are under-represented in academic research. Hearing about the lived experiences of these groups encourages more inclusive planning and earlier consideration of their research priorities:

*It [a CoREN event] was in the area of working and researching with Traveller and Gypsy and Roma communities*,* and the reason it was so informative is that most of the sessions were presented by people from those communities… it’s really something that’s stayed with me. Just how very excluded those communities are from everything*,* let alone research. So*,* whenever there’s an opportunity to feed into people’s plans for research*,* I will say “have you thought about that community?”. (Researcher interview 2)*

As a boundary-spanning space, the CoREN gives greater voice to communities and, by bringing researchers into first-hand contact with diverse groups, helps reduce researchers’ biases that might otherwise persist through lack of grounding in lived experience:

*What I’m saying is*,* 6*,* 7*,* 8 years of academia can shape your thinking to a way that isn’t the same as somebody who’s in a very deprived socioeconomic environment. So*,* CoREN is a brilliant vehicle to bring these people together. It benefits the researchers… by going into communities and understanding some of the issues they face. (Leadership Group interview 2)*

#### Building communities’ research literacy and capacity

Participant accounts provided details of how the CoREN spreads research literacy, demystifying research and challenging stereotypical views of it as a distant activity, with minimal opportunity for VCFSE organisations to contribute. Raising research literacy can put community partners on an equal footing with academics to take part in co-production [24], with, for example, the following non-academic participant welcoming the opportunities the CoREN had given them to learn about mental health, catalysing their further involvement in research such as data collection:

*I got to know that children also have mental health issues*,* not only the adults*,* and that’s why I got involved more in ARC NWC and got to know more about other things as well… I’ve been involved in focus groups*,* workshops*,* events*,* conferences… I got to know more about so many things. (Focus group 3 - Participant 4)*

By gaining research knowledge, the Leadership Group member below revised their views of academic research, and came to appreciate that her organisation possessed a great deal of privileged insight that researchers would struggle to access without working in partnership with VCFSE organisations and members of the public:

*The sort of work that we do*,* we don’t see it as research. I remember when I heard the term research*,* I always used to think it was a lab and somebody in a white coat. But the wealth of information that we have. And an academic coming into our community*,* there’s no way they would understand*,* and they don’t know. (Leadership Group interview 4)*

The growth in community research awareness had benefits for personal and professional development, with individuals gaining skills to translate ideas into researchable questions, learning how to navigate and access research funding, and, through the CoREN, becoming more effective agents within formal research studies:

*For my personal development*,* I’ve learned loads as to how the academic system works… Through wanting to do my research that I’m doing now*,* and if I wasn’t involved with ARC*,* or I wasn’t involved with CoREN*,* these light bulb moments would have never occurred to me… Those questions come to my head and I can put a question to it… I’ve got learnings that I can put into bids… you know the buzzwords… it put me in a better position for certain funders. (Leadership Group interview 4)*

From the point of view of the regional healthcare system as a whole, there are benefits to communities gaining research literacy through engagement with the CoREN as they are better placed to engage in broader research conversations about system change:

*I think research and the power of enquiry and supporting people to develop a curiosity and to develop a sense of their own capacity*,* I think all of those things are helpful in skilling people to engage in that dialogue that we need to have about what we want from a system that we all contribute to… Things like CoREN are sowing the seeds of a future that will enable those dialogues to take place because they’ll be more informed. (Leadership Group interview 3)*

#### Evidence of deliberative and relational dynamics within the CoREN

Encouragingly, participants’ accounts of the CoREN often echoed the democratic and relational principles that underpin its goals. For example, the participant below linked flattening of traditional hierarchies to the creation of unexpected opportunities and to changes in research practice and in who sets its agenda. The generation of unexpected research opportunities echoes the case made in relational approaches to co-production evaluation regarding the value of unpredictable outcomes [14] that can result:

*Having spent an awful lot of time in academia*,* it’s really nice to have that collegiality*,* to have that openness and sharing*,* and that creates opportunities for everybody and it kind of levels the playing field a little. (Researcher interview 1)*

This change in status and negotiation of hierarchy was commented on elsewhere by a member of the Leadership Group, who, using language that hinted at a bridging between scientific expertise and lived experience, pointed out that researchers depend on community insights. They implied this reliance had given community members confidence and security that their presence is valued:

*I think you guys need us… even if it’s really scientific*,* you still need that community involvement*,* and that’s how the community can get involved and support. (Leadership Group interview 4)*

As well as their intrinsic value, relational and democratic dynamics were valued for their instrumental power as a means of generating the “right” research questions through empowering communities to influence strategic priorities. In this case by creating a bridge between communities and health systems:

*I don’t think we can ask the right questions without that level of community engagement and through doing that*,* you kind of empower as well. I think organisations like CoREN enable us to have conversations that feed into the agendas of the ICB*^7^, *that feed into the wider strategy to ensure that we’ve got the voice of the community. (Researcher interview 1)*

Consistent with deliberative theory, empowering participants by giving them control over the research process changes how they see themselves, heightens their investment and makes them identify more closely with the work. The participant below made this point by drawing a contrast with superficial engagement that begins and ends with involvement in data collection:

*It’s actually having the chance to say I want to do this piece of research… following it through all the way… And then you feel it’s really your project*,* rather than somebody coming from outside saying “we’re doing research… let’s ask you a few questions” and then they disappear. (Focus group 3 - Participant 2)*

Disputation and the continuous testing of claims are key aspects of deliberation that give voice to diverse, often overlooked knowledge within communities and VCFSE organisations and bring to light priorities that might otherwise be missed, enabling researchers to use that knowledge when setting research priorities. By exposing participants to new information, deliberation gives both community members and academics the opportunity to revise their views. One researcher recalled a session in which deliberative processes challenged assumptions about where research priorities should lie:

*It highlighted the sheer number of people that make up that community and that what we think is a priority actually isn’t. So*,* there’s an exercise that we did where we all kind of looked at what would our priorities be*,* what would those questions look like? And the difference was amazing. (Researcher interview 1)*

On a similar note, another example of how deliberative dynamics could lead to tangible change was the evolution of the CoREN’s mentoring programme. The initiative was initially conceived as “reverse mentoring,” a model where community members would mentor researchers. However, participants identified that this framing inadvertently reinforced a binary hierarchy by implying a one-way flow of learning where researchers lacked ‘lived experience’ while community members provided it:

*It started off as reverse mentoring… that project had been seen initially as though community members within the CoREN would be mentors for researchers*,* but we were kind of saying that as researchers*,* we also have lived experience of things as well. (Researcher interview 3)*

During preparatory workshops designed to shape the programme, participants tested these claims. Through a process of mutual listening, the group deliberated on the principle that learning should be reciprocal rather than one-way, moving away from a hierarchical “mentor/mentee” relation towards a partnership of equals:

*We did one or two [workshops] which was shaping what we thought reverse mentoring should be and boundaries… approaches and so on about being a like mentor or mentee. (Leadership Group interview 2)*

*When we got into the discussions about it*,* we were reflecting collectively as a group that actually it’s more about collaborative mentoring. (Researcher interview 3)*

As a result of this deliberation, the initiative was renamed and rethought, replacing a hierarchical model with an egalitarian space for knowledge exchange.

Respecting community influence within deliberative processes of this sort is key to the legitimacy and success of deliberation [25]. Given this, a Leadership Group member pointed out that, because VCSFE representatives sit on the Leadership Group, the CoREN can act as a guarantor of community interests and influence within research networks, guarding against extractive use of community knowledge and helping communities to benefit meaningfully:

*It’s [the CoREN] kind of like a protector of community groups and they’re not used and abused for their knowledge and for their experiences… I’m a member of CoREN… as like a kind of community leader within the thing I would be that protector*,* so to speak*,* and I would make sure that the community gets something out of it as well. (Leadership Group interview 6)*

### What is driving the successes of the CoREN?

#### Collaborative, committed leadership and collective decision making

Participants pointed to leadership as a key driver of the CoREN’s impacts discussed above. In particular, the SEM was praised for skilfully negotiating the delicate balance between investing personal energy in guiding the network’s development while resisting the urge to centralise control to hasten change. Given the CoREN’s commitment to shared decision-making and the need for a leadership style that supports the deliberative processes described above, managing this balance is crucial:

*I’m thinking of [the SEM]…she wants to sort of drive it forward*,* but also democratise it and make it a network*,* as the name suggests*,* and that’s really difficult. You know*,* that’s a real skill to do that because… an individual*,* a figurehead*,* actually becomes a bit of a default leader*^8^, *but you don’t get that feeling that she is directing this. (Researcher interview 2)*

As we see in the quotation below, this balanced leadership approach is underpinned by a mindset of “always questioning”. A willingness to reflect, learn, admit mistakes and invite other perspectives fits well with a network built on inclusion and the equal legitimacy of diverse perspectives:

*She [the SEM] is very approachable… She’s always questioning… “what is the role of the CoREN*,* what’s the kind of best way that we can work with this project?” (Leadership Group interview 6)*

*They’re learning experts. They’re developing experts…They haven’t sorted this sphere. And I think they’re very open to hearing about other experiences and learning…They know they haven’t got all the answers*,* and they haven’t perfected public involvement. They have the intention and the integrity to that principle and the values of participation. (Researcher interview 2)*

This open and curious approach is fundamental to the success of the CoREN as it operates in a field in which best practices are not well known or established. It also complements the CoREN’s egalitarian model of collaboration, creating an inviting platform for participation by signalling that the leadership is keen to learn from others and aware of its own fallibility. This role for democratic leadership in energising the network is echoed in the wider literature, which links stronger outcomes to “leaders who advance a democratic orientation” [14] and promote inclusive and deliberative practices [14, 26].

### Challenges for the CoREN

#### Clarity of the CoREN’s role

Among the factors that may be inhibiting greater success for the CoREN, a clear challenge was noted with understanding of the CoREN’s structure and purpose, including its relationship with the ARC NWC. Participants linked difficulties understanding the CoREN to shortcomings in communicating why stakeholders would want to be involved, and how:

*From a voluntary perspective*,* why would we want to be involved in CoREN? What good is it gonna do us would be the question I would ask. If I get any emails saying you need to be part of this*,* well*,* why? So*,* understanding that from a sector perspective is vital and people are confused about the differentiation between CoREN and ARC and comms [communications] is definitely [important]. (Focus Group 1 - Participant 5)*

#### Lack of inclusivity

Despite the CoREN’s success with facilitating diverse collaboration, there was awareness among interviewees that it was still engaging more with accessible, privileged populations, with challenges remaining with penetrating into seldom-heard, less privileged communities that might benefit the most from engagement with researchers. For instance, the quotation below shows the researcher’s desire for the CoREN to facilitate engagement with a more diverse range of participants than they are typically able to involve. They felt that the CoREN could be doing more to enable this broader engagement with seldom-heard communities, suggesting that it could create a centralised and accessible database of under-represented community groups:

*I guess one of the tests for me was “I need some help setting up some public engagement workshops… can anyone help me get in a room with people?” And it failed that test because no-one came forward… Do we have a little map where there’s pins on it… this is a Muslim ladies group*,* this is an old age pensioners thing*,* this is a disability group… That is the thing that I think it [the CoREN] should be delivering… And then researchers will find more value because it’ll be like “these are the people to go to”. For me it’s really about challenging ourselves to build the layer down*,* of even less well heard people. (Researcher interview 4)*

A further aspect of the CoREN’s challenge in connecting the region’s diverse communities relates to its geographical reach and the significant challenge of achieving a presence across several large urban centres and rural communities. As the CoREN seeks to spread awareness and broaden its network of contacts throughout the region, some participants warned that this aspiration was not adequately reflected in geographical distribution of collaborators or the Leadership Group:

*In the Leadership Group*,* or with who you’re working with*,* are all areas covered? Because it might just all be located in one area and they’re receiving all the benefits. But what about the other areas? (Focus Group 1 - Participant 6)*

Participants also felt that the network was not making adequate use of community venues for its events. The researcher quoted below questioned if current practices in this regard truly embody a commitment to community engagement:

*Is CoREN supporting those smaller organisations to host the little events? You wanna get to the heart of the community. Why are we not using the art galleries? Are we using the cafes? Are we using the scout club? Are we using the local schools?… making sure that we are going to people rather than people coming to us. (Researcher interview 1)*

#### Structural and organisational challenges

Although the SEM was praised for their collaborative leadership style, there remained a perception among some participants that the CoREN could share control better if it were less hierarchical. This was expressed less as a criticism and more as an aspiration that the CoREN try to reach even greater levels of egalitarianism and control by community representatives:

*How can they become more egalitarian perhaps in their leadership? I know that there’s the Leadership Group*,* but maybe there is room for an increase… maybe they could go further by maybe having a co-leadership role to demonstrate a more collaborative approach because there’s still that hierarchical impression. (Researcher interview 1)*

While the participant from Researcher Interview 1 above expressed a clear preference for greater democratisation, below they acknowledge the intrinsic and structural factors that impede the CoREN’s progress as a network that straddles the worlds of community activism and formalised bureaucratic structures:

*It’s really hard because inevitably it’s going to be a hierarchical organisation*,* I guess because of where it sits within big organisations and institutions…It’s a real tricky one because as soon as you’ve incorporated community activists in and formalised it*,* you’ve changed it. (Researcher interview 1)*

Alongside calls for greater democratisation, inclusivity and reduced hierarchy, there was also a somewhat contradictory suggestion made that deliberative decision-making processes within the CoREN could obscure responsibilities and increase bureaucratisation, making it challenging to efficiently link researchers with community groups. The academic interviewed in researcher interview 4 contrasted the organisational structure of the CoREN with the Blackpool Health Determinants Research Collaboration (HDRC), noting that the HDRC was more able to help with their requests because it was clear who was responsible for connecting researchers to specific communities and organisations.

*If you go to a specific group or person*,* like the head of that organisation or person who owns that patch and you speak directly to them*,* they’re much more likely [to help]… they’re [the HDRC] more likely to help you out because they can’t diffuse into a mass of no accountability*,* because you’re literally asking them. So go to HDRC. I’m asking an individual or a couple of individuals to help straight away. (Researcher interview 4)*

This suggests a possible tension between democratisation and the need for responsive, transparent and accountable leadership that can accelerate decision making and minimise delays. Such a trade-off is not specific to the CoREN and is an ongoing dilemma for all initiatives that adopt structures and leadership styles that genuinely support power sharing.

### How can the CoREN improve?

As well as identifying impacts, factors underpinning success, and challenges for the CoREN, participants proposed strategies it could employ to fuel further progress and address its challenges. While there was consensus that the CoREN had been successful in deepening collaboration between communities, practitioners, and researchers, it was recognised as an ongoing and unfinished endeavour. To further deepen these collaborations, participants suggested facilitating greater community involvement in every phase of research projects. This was thought to be important because tasks such as data collection and data analysis are conventionally seen as the preserve of academic researchers, representing a sphere where public involvement is typically minimal, and the power of researchers goes unchecked:

*Where it has worked so far is getting people involved in the start of a concept and them helping to share what the outcomes of the work are. But that middle part of co-producing the actual research…I think there is scope for them to be involved in helping teams to shape methods. It feels like it’s just connecting the dots because you’ve got them involved in the start… and you’ve got them involved at the end… But you’ve not got them involved in the middle to kind of like connect that thread. (Researcher interview 3)*

At a more strategic level, participants indicated that the CoREN could strengthen its non-project-bound model of collaboration by deepening community influence on future research priorities. The member of the Leadership Group quoted below stressed that the CoREN should prioritise community-originating research, reversing the dynamic in which a researcher uses the CoREN to gain community involvement for research projects that are already well underway:

*As a researcher*,* if you’re looking for community involvement*,* come and talk to the CoREN and we’ll find you somebody. It doesn’t feel like it goes the other way*,* so I think that’s where it could go… It feels to me like community origination of research… could be a really good outcome of the CoREN… If there’s a community that wants research doing*,* how do we bring research capacity to that? (Leadership Group interview 5)*

Providing practical suggestions as to how the CoREN could further facilitate connections between researchers and communities and provide a means to capture community-driven research ideas, the following participant suggested researchers’ time could be set aside to regularly work with VCFSE organisations on their research idea:

*One of the things (VCFSE organisation member) and others have talked about potentially is maybe having an academic*,* once a week*,* and say “Look if you think you’ve got something that you want to dig into and learn a bit more or do research*,* you know you’ve got access to an academic who can support you”. (Leadership Group interview 4)*

## Discussion

Overall, the evidence presented above suggests the CoREN is functioning well as an intermediary, bridging gaps between diverse community, practitioner and research stakeholders. It has promoted greater community involvement in research and has increased research literacy in those communities, thereby strengthening their capacity to contribute to research. However, challenges remain, particularly regarding understanding of the CoREN’s role, lack of inclusion of communities in the region, over-reliance on leaders, and bureaucratic inertia. These findings point to strong progress and provide a plausible basis for extending and adapting the model beyond the North West Coast region, but also to the need for further support if the CoREN is to exploit its potential in more geographical and social niches.

### The CoREN as a facilitator of partnership synergies

The CoREN’s progress in its role as an intermediary suggests it has begun to play a key role in boosting collaboration in health and social care research in the North West Coast region. Its role can be seen as facilitating partnership synergies [27], meaning the improved research outputs that result from organisations, communities, and individuals pooling their perspectives and resources rather than working alone. The notion of partnership synergy offers theoretical backing for the intermediary role the CoREN plays as it enables collaboration between otherwise unconnected partners. With its calendar of community-based activities, shared leadership structure, and sustained model of collaboration, the CoREN appears to be well placed to maintain these synergies, particularly as these same factors are said to promote long-term community engagement in research and increase the scope for systemic impact [28, 29].

Theory suggests it is important that the CoREN is maintaining these conditions over a longer term. For instance, Ansell & Gash [30] show it is the ‘virtuous cycle’ of incremental improvement, deepening trust and shared understanding that gradually builds social capital [31]^9^, shared frames of reference, and institutional capacity. The CoREN’s sustained model of engagement is therefore conducive to generating enduring synergies and the cumulative cultural change needed to dislodge practices and norms that run counter to co-production.

In this light, participants used a metaphor of “planting seeds and watching the tree grow” to describe the CoREN’s contribution. This reflects established theory about how collaborative relationships build capacity and unforeseen opportunities beyond initial contact [32, 33]. As such, the real dividend of the CoREN’s work may be the durable social capital it generates through shared trust, networks, and cultural changes that continue to contribute to innovation in ways that could not be predicted at the inception of the network [32, 33].

### Incremental progress and structural constraints in democratic knowledge production

We have seen that the CoREN’s practices reflect an effort to democratise decision-making and share power. Our analysis uncovered promising signs that the CoREN’s work includes elements central to deliberative democracy such as negotiation of status hierarchies and creating spaces in which community voices were valued and depended upon. There was also evidence of distinctively deliberative processes of mutual listening, testing of competing claims and revision of views. In relational terms, ongoing inclusion and empowerment of communities through sustained relationships enabled them to exercise more agency, with participants describing feelings of investment and identification with the CoREN as their own co-creation. The shared authority and decision making that result adds weight to claims of democratic legitimacy for the network.

Other practices we identified that promote democratisation included working with communities and professionals to build their research literacy, including mechanisms such as the Leadership Group, and Collaborative Mentoring sessions where researchers and community members exchange knowledge and ideas on an equal footing. These features of the CoREN reflect a commitment to treating community representatives as equal partners in the research process. This is welcome because sustained commitment to principles of shared governance and reciprocal exchange of knowledge are seen as key to successful co-production and user participation [27, 34].

Alongside these successes, our findings suggest that while the CoREN has improved access for many, there remains a gap between the ideal of egalitarian collaboration and the current level of progress. Participants voiced concerns with jargon that may serve to deter possible partners if they do not know what the CoREN does or how it links to the ARC NWC; the clustering of face-to-face events in a few locations; and the need for more community control over the earliest stage of research development.

The significance of these challenges should not be underestimated, with others finding that similar problems [30] such as poorly defined roles, participants that don’t reflect the target community, and lack of involvement throughout the research cycle can prevent participation moving beyond tokenism. What may seem like merely logistical oversights, such as venue choice, can affect progress towards democratic goals of reducing power imbalances [35] and obstruct progress towards the transformative potential of shifting who sets research questions [36]. Similarly, unfamiliar, inaccessible spaces, or formal styles of interaction may deter marginalised voices, while informal, community-based venues have been shown to encourage broader, more equal participation [37, 38]. Such is the potential significance of these challenges that the CoREN should systematically and forensically review the details of its outreach to communities.

Part of reaching out more successfully and understanding the needs of communities is building awareness within the CoREN of structural inequalities that can hamper progress, as has been noted for similar initiatives [35]. Only when these inequalities are understood at the local level can the work of the network begin to overcome their pernicious influence on efforts at democratisation. More immediate practical measures to offset the impact of structural inequalities could involve greater material support to increase the control exercised by a broader base of stakeholders, and for equitable outreach and engagement.

Among those structures preventing wider participation are the rigid and traditional conventions found in academia. The observation that jargon and geography can exclude potential participants reminds one that even in a well-conceived co-production initiative such as the CoREN, efforts to democratise research are liable to run up against forces that lie well beyond the control of any single researcher or public contributor. For example, at the funding stage, bodies such as the NIHR still judge proposals primarily by conventional criteria such as a track record of publications, previous grants and recognised methodological expertise [39]. Though there are other criteria intended to incentivise public involvement, arguably, primary criteria still reproduce the hierarchies that initiatives like the CoREN are designed to challenge. Without top-down institutional action to counteract these structural challenges, countervailing bottom-up efforts by the CoREN and its members will remain constrained in the success they can achieve. This is not a counsel of despair but a recognition that exhorting the CoREN and its members to do better has its limits when powerful incentives continue to reward other priorities ahead of co-production.

## Conclusion

Reflecting on the implications of our work for those building or assessing similar networks, there is a methodological challenge in tracking and attributing specific outcomes or research projects to relationships formed within a diffuse network. The process through which relationships incubated within the network contribute to downstream impacts is mediated by a huge range of other factors, and the results of the network’s work may emerge slowly and indirectly, making them difficult to observe or quantify. Those starting or evaluating comparable initiatives should not take this to mean that immaterial and relational impacts are any less significant. Indeed, we urge them to look beyond short-term, easily measured results towards the intangible, longer-term processes that give networks their distinctive worth, and, in doing so, to help push academic research towards recognition of this long-term, relational framing.

Reflecting on the implications of our work for the CoREN itself, we have seen the promise offered by its model of sustained investment and structures that promote communities’ capacity to shape longer-term research processes. However, its progress towards community inclusion is uneven, and with more time comes the tendency for pervasive patterns of inequality and social power structures to undermine progress. It is vital therefore that the network redoubles its efforts to reach all communities, forming stronger roots in the community that cannot easily be uprooted by growing inequalities or creeping institutionalisation. As the CoREN continues to seek to deepen its democratic legitimacy and representation of communities, it is salutary to remember that mere expressions of support for deeper co-production can obscure whether these goals are being achieved [40, 41]. Continued investment, robust evaluation and democratic internal feedback mechanisms are needed to ensure the CoREN can reach its considerable potential.

## Strengths and limitations

A limitation of this evaluation is that it was conducted internally by employees of ARC NWC, the funder of the CoREN, which may increase the risk of a favourable bias towards the network. Convenience sampling, recruitment through existing CoREN relationships and partner networks, and voluntary uptake may also have favoured participation by individuals who were already engaged with, and positively disposed towards, the CoREN. Another limitation is that the findings may be context-specific to North West England, and generalisability beyond the region may be limited. Additionally, to effectively capture downstream impacts of the CoREN, more sustained and longitudinal evaluation methods might have been more effective. For example, the CoREN logic model anticipates a quantifiable increase in public-led research projects, and thus developing a feasible indicator and routine monitoring approach for this outcome would be a useful component of future longitudinal evaluation. In the future, this could be combined with social network analysis to map the connections formed through the network and their contributions to the broader research ecosystem.

The sample in this evaluation presented both strengths and challenges. Including most of the Leadership Group in the interviews allowed for a rich understanding of their perspective. However, we had limited participation from ARC NWC research staff, which may have constrained the evaluation’s ability to fully capture the scope of research work undertaken as a result of the CoREN’s activities. This is significant because co-production depends on sustained involvement from both public contributors and researchers. With only partial input from research staff, our account of the academic side of the partnership is incomplete.

We also did not collect participant demographic data or organisational identifiers in order to protect confidentiality in a small network. This limited our ability to assess the diversity of the sample, which is important given the CoREN’s aim to include marginalised groups and address health inequalities. It also limited our ability to examine how intersecting structural forces (for example gender, race, class, disability, migration status, and language) shaped participation in the CoREN and in this evaluation. While the CoREN aims, in broad terms, to address structural inequality by increasing inclusion in research, our methods did not allow us to examine systematically who was included or excluded, and why. In addition, because our analysis focused on relational dynamics and did not assess the influence of structural conditions (for example socio-economic position or experiences of discrimination), our conclusions about inclusion are limited to the relational and organisational level. Addressing macro-structural mechanisms of exclusion and power would require other data and methods and is an important priority for future evaluation.

Key strengths of this evaluation are that it has brought to light successes and areas for improvement, providing practical guidance for the CoREN’s future development. It has also raised the profile of an innovative and sustained regional model of co-production and introduced some principles that will be of use to others leading or evaluating other such initiatives. Lastly, its theory-informed approach shows how the CoREN’s processes relate to concepts such as relational evaluation, deliberative democracy, structural and power constraints, and the diffuse, mediated impacts of facilitative co-production activities. In doing so, it presents an adaptable theoretical framework that can inform future evaluations of other sustained collaborative research networks.

## Supplementary Information

Below is the link to the electronic supplementary material.

Supplementary Material 1

Supplementary Material 2

Supplementary Material 3

Supplementary Material 4

## Data Availability

The interview and focus group transcripts contain potentially identifiable information, and participants did not consent to public sharing. The data are therefore not publicly available due to privacy and ethical restrictions. De-identified excerpts supporting the findings are included in the article, and the topic guides and coding framework are provided in the Supplementary Files. Additional data may be available from the corresponding author on reasonable request.

## Footnotes

1 By ‘democratic co-production’ we mean practices that create inclusive spaces for reason-giving and mutual listening; share agenda-setting and decision-making between researchers and stakeholders; treat experiential and professional knowledge as equally indispensable; and sustain relationships beyond single projects.

2 Here, ‘ARC NWC members’ refers to ARC NWC’s formal membership across partner sectors (including NHS, local authority, and university partners). This is distinct from the wider audience who may attend ARC events or otherwise have ad hoc contact with ARC NWC.

3 These are VCFSE partners contracted and funded by ARC NWC to deliver CoREN activities and to sit on the CoREN Leadership Group.

4 Open membership refers to the inclusive network of community groups, NHS or local authority staff, academics, or members of the public interested in the CoREN’s mission who have attended events or joined the CoREN mailing list, who could potentially act as research collaborators or as part of generating research priorities. These members do not have defined obligations.

5 For example, Greenhalgh et al. [14] identify five “health research systems” or “multi-stakeholder research collaborations”. They use these terms to refer to organised networks with formal structures and governance that bring researchers and service users together early to co-produce locally relevant research and support implementation.

6 Such an approach is consistent with many of the core elements set out in NIHR’s Public Partnerships Strategic Commitments Logic Model [1], including those concerned with strengthening partnerships and building public involvement capability and capacity.

7 ICB refers to Integrated Care Boards, the statutory NHS body for each Integrated Care System, created by the Health and Care Act 2022, which plans and commissions local NHS services.

8 In practice, this ‘lead’ role sat alongside the Leadership Group’s strategic oversight. As well as providing day-to-day coordination and supporting delivery, the SEM developed ideas through informal conversations within the network and then took them to the Leadership Group for discussion, refinement, and agreement before implementation.

9 Social capital refers to the networks of relationships and trust that individuals are embedded within, and the benefits and resources people gain from their social networks and relationships. The CoREN facilitates ‘bridging’ social capital by connecting disparate groups (researchers and community members) who would not otherwise interact.
