# Supplementary material for: Democratising health and social care research through long-term public involvement and engagement: a qualitative process evaluation of the Community Research and Engagement Network (CoREN)
Source: Res Involv Engagem. 2026 May 23;12:70. doi: 10.1186/s40900-026-00897-2 (PMC13198050; doi:10.1186/s40900-026-00897-2)
Supplement: Supplementary file 1 — Supplementary Material 1 [file 40900_2026_897_MOESM1_ESM.docx]

# Evaluation of the CoREN – List of Codes used in Qualitative Analysis

| Name |
| --- |
| Barriers to community involvement in research (not pertaining to the CoREN) |
| Community organisation's experiences of barriers to community involvement |
| Leadership group perceptions of barriers to CI |
| Professional's experiences of barriers to community involvement |
| Researcher's experiences of barriers to community involvement |
| Barriers to participation in the CoREN |
| Barriers to research participation (not pertaining to the CoREN) |
| Barriers to the participation of communities or individuals in research |
| Barriers to the participation of community organisations in research |
| Barriers to the participation of members of the leadership group in research |
| suggestions to help address barriers |
| Challenges working with and within the CoREN |
| Community organisation's challenges working with the CoREN |
| Members of the leadership group's experiences of challenges working within the CoREN |
| Researcher's challenges working with the CoREN |
| Changes in research activity post contact with the CoREN |
| Community Organisations - changes in research activity post contact with CoREN |
| Members of CoREN leadership group - changes in research activity post contact with CoREN |
| Researchers - changes in research activity post contact with CoREN |
| Changes to research culture or attitudes after contact with the CoREN |
| Changes in culture or attitudes pertaining to research |
| Pre CoREN culture or attitudes pertaining to research |
| The CoREN’s role in promoting connecting, coordinating and networking |
| community organisations |
| Leadership group |
| researchers |
| Impact of the CoREN on relationships between research staff and VCFSE organisation staff |
| Impacts of the CoREN on community involvement in research |
| Impact of the CoREN on community engagement skills |
| Impact of the CoREN on the degree and nature of community involvement in research |
| Impacts of the CoREN on interest in involvement with the ARC NWC |
| Impacts of the CoREN on interest of community organisations in being involved with the ARC |
| Impacts of the CoREN on interest of members of the leadership group in being involved with the ARC |
| Impacts of the CoREN on interest of professional researchers in being involved with the ARC |
| Impacts of the CoREN on participation in research |
| Impacts of the CoREN on the interest of members of community organisations in participating in research |
| Impacts of the CoREN on the interest of members of the leadership group in participating in research |
| Impacts of the CoREN on research (not directly concerned with CI) |
| Impact on research carried out by LG |
| Impacts of the CoREN on research carried out by community organisations |
| Impacts of the CoREN on research carried out by professional researchers |
| Research literacy and understanding |
| Leadership group |
| Expectations |
| general aspirations |
| Leadership group role, activities undertaken |
| Levels of influence, agenda setting, ownership |
| LG – what is not working well |
| LG – what is working well |
| LG Perceived role of the CoREN in Coproduction |
| Notable quotes |
| Other impacts of the CoREN |
| Perceptions of ARC NWC |
| Community organisations perceptions of ARC |
| LG group perceptions of ARC |
| Researcher perceptions of ARC |
| Perceptions of community involvement in research |
| Community organisations and community members perceptions of community involvement in research |
| CoREN leadership group perceptions of community involvement in research |
| Researcher perceptions of community involvement in research |
| Perceptions of the CoREN |
| challenges and considerations for the CoREN |
| Not working well in CoREN |
| should do or do more of |
| Working well in CoREN |
| Research Practices pre contact with CoREN |
| Community organisation's research activity occurring independently of contact with the CoREN |
| Members of leadership group - research activity prior to contact with the CoREN |
| Researcher's research activity occurring independently of contact with the CoREN |
| Understanding of the CoREN and its role |
