# Supplementary material for: Democratising health and social care research through long-term public involvement and engagement: a qualitative process evaluation of the Community Research and Engagement Network (CoREN)
Source: Res Involv Engagem. 2026 May 23;12:70. doi: 10.1186/s40900-026-00897-2 (PMC13198050; doi:10.1186/s40900-026-00897-2)
Supplement: Supplementary file 2 — Supplementary Material 2 [file 40900_2026_897_MOESM2_ESM.docx]

# Evaluation of the CoREN - Topic Guides

The topic guides below were used flexibly to provide a loose structure to the interviews and focus groups, allowing researchers to adapt to the priorities of participants. They were developed with members of the CoREN.

**Interviews with CoREN Leadership Topic Guide**

- Perceptions on current research practice with VCSFE organisations
- Perceived benefits of community engagement and co-production in research
- Perceived benefits of being engaged/involved with the CoREN
- Understanding, experience and awareness of research and evaluation methods
- How else they have been involved with the CoREN (if any)
- How involvement in CoREN has influenced research and community engagement skills
- How involvement in CoREN has influenced motivations and confidence to get involved/conduct research.
- Research practice/culture (within member work roles/organisations-pre and post CoREN involvement)
- Opinion on facilitators / barriers to successful co-production
- How has/should the CoREN contribute to co-production
- Organisations they have worked with and who they wish to work with in future
- Control/ownership of the CoREN
- Challenges of working with the CoREN
- Recommendations for future working of the CoREN

**Interviews with Researchers who have engaged with CoREN Topic Guide**

- Research practice/culture in current research roles/organisations (pre and post CoREN involvement)
- Perceived benefits of community engagement and co-production in research
- Perceived benefits of being engaged/involved with CoREN
- How they have been involved with the CoREN
- How involvement in the CoREN has influenced their research
- Experience of engaging with the CoREN and members
- Facilitators / barriers to successful co-production
- How has/should the CoREN contribute to co-production
- Other experiences of co-production (and how this is similar/different to CoREN)
- Challenges of working with the CoREN
- Recommendations for future working of the CoREN

**Focus Group with COREN members Topic Guide**

- Perceptions of current research practice with VCSFE organisations
- Perceived benefits of community engagement and co-production in research
- Perceived benefits of being engaged/involved with the CoREN
- Understanding, experience and awareness of research and evaluation methods
- How they have been involved with the CoREN
- How involvement in the CoREN has influenced research and community engagement skills
- How involvement in the CoREN has influenced motivations and confidence to get involved/conduct research.
- Facilitators / barriers to successful co-production
- How has/should the CoREN contribute to co-production
- Control/ownership of CoREN
- Challenges of working with the CoREN
- Recommendations for future working of the CoREN
