# Supplementary material for: Democratising health and social care research through long-term public involvement and engagement: a qualitative process evaluation of the Community Research and Engagement Network (CoREN)
Source: Res Involv Engagem. 2026 May 23;12:70. doi: 10.1186/s40900-026-00897-2 (PMC13198050; doi:10.1186/s40900-026-00897-2)
Supplement: Supplementary file 3 — Supplementary Material 3 [file 40900_2026_897_MOESM3_ESM.docx]

**1. Aim**
We involved public contributors so the evaluation asked the right questions, used clear and accessible materials, and produced findings that communities and practitioners could use. We involved a public adviser (HG) and sought feedback from the CoREN Leadership Group, composed of representatives from commissioned VCFSE organisations. HG had input into study design and data-collection plans, materials and question wording, analysis, and reviewed manuscript drafts to ensure the write-up was clear. The Leadership Group sense-checked emerging findings to guard against blind spots of the research team and to help frame recommendations in terms that are meaningful to those involved with the CoREN.

**2. Methods (how involvement happened)**

A small steering group for the project (n=4) included one public adviser (HG). She joined early design meetings, reviewed data collection instruments, and attended several meetings across the project. HG’s involvement was mainly via meetings and comments on draft documents. We did not keep a full log of her input, which limits precise attribution of changes to her input. Her activities included:

- Input on overall study design, aims/objectives; review of interview/focus-group question topic guides; comments on participant materials; practical input into planning and set-up of focus groups.
- Supported planning for focus groups, but, due to personal circumstances, stepped back before most primary data collection and did not participate in it herself.
- Reviewed preliminary findings and manuscript drafts; suggested deletions of superfluous text and edits to improve fluency/clarity; confirmed lay readability.

We also presented emerging findings to the CoREN Leadership Group, seeking verbal feedback at the time and inviting written responses.

**3. Study results**

**Positive changes attributable to PPI:**

- Practical advice on set-up and conduct of focus groups, including planned topics/questions.
- Improvements to language and readability, including trimming superfluous material; clearer phrasing and flow in the manuscript; confirmation that the text was accessible to a non-academic audience.

Sense-check of findings and recommendations (Leadership Group):

- Need for clearer communications and fewer acronyms; confusion about ARC/NIHR/CoREN relationships.
- Value of locally based “Collaboration Cafés” but more needed across multiple towns, including smaller settlements to widen reach.
- Importance of involving commissioners in events.
- Desire for a central online space for CoREN outputs (searchable by theme/location).
- Support for resourcing community organisations (e.g., reimbursement for hosting researchers/training community members).

**Shortfalls/negatives:**

- Involvement was intermittent during intensive writing periods.
- Record-keeping was incomplete, so some specific changes cannot be traced to a named suggestion.

**4. Discussion and conclusions**

Overall, there was a well-embedded level of public influence. Involvement of HG and the Leadership Group strengthened the clarity and practical relevance of recommendations (especially on communications, event location, and resourcing). The Leadership Group had limited influence on methodological choices taken during earlier stages of the project. They were, however, kept informed throughout and invited to comment on progress, and their feedback indicated they were broadly content with the approach taken. HG had ongoing input into study design, data collection and data analysis discussions.

**5. Reflections/critical perspective (what went well / what to change next time)**

We are satisfied that HG was involved in early discussions of study design, continuing to be involved and offering feedback on data collection tools and drafts that was acted upon. HG reports that her suggestions were “heard, discussed and acted upon,” and where not adopted, reasons were explained. The Leadership Group’s sense-checking of findings affirmed their resonance with the experiences of the group and led to changes in emphasis in reporting of findings.

We have learnt that it is better to maintain an ongoing log detailing specific PPI inputs and their impact. In future, we will also plan more time for public involvement during more intense periods of writing. To improve equity and inclusion, more resources could be used to broaden engagement beyond those already known to the researchers, reducing barriers to involvement.
