# Supplementary material for: Democratising health and social care research through long-term public involvement and engagement: a qualitative process evaluation of the Community Research and Engagement Network (CoREN)
Source: Res Involv Engagem. 2026 May 23;12:70. doi: 10.1186/s40900-026-00897-2 (PMC13198050; doi:10.1186/s40900-026-00897-2)
Supplement: Supplementary file 4 — Supplementary Material 4 [file 40900_2026_897_MOESM4_ESM.docx]

**ARC NWC (Applied Research Collaboration North West Coast)**: A regional partnership funded by the NIHR that brings together universities, the NHS, local authorities, and other partners to conduct applied research. It funds and hosts the CoREN.

**Boundary-spanning space**: A description of the CoREN as a space that enables collaboration across boundaries that often prevent groups from working together (e.g., communities and academics).

**Capacity building**: Developing the skills, confidence, relationships, structures and resources that enable people, organisations and communities to take part effectively in research and to sustain that involvement over time.

**CLAHRC (Collaboration for Leadership in Applied Health Research and Care)**: The predecessor NIHR organisation to the ARC NWC. The CoREN’s origins lie in the CLAHRC’s Neighbourhood Resilience Programme (2016-2019).

**Collaboration Café**: Periodic events organised by the CoREN that mix short talks with table discussions to help "seed" projects.

**Collaborative Mentoring**: A CoREN activity pairing researchers with VCFSE partners in reciprocal roles for two-way learning.

**Co-production**: A collaborative form of involvement based on equal and sustained partnerships between researchers and the public, involving shared power and joint decision-making throughout the research cycle.

**Coordinating nexus**: A description of the CoREN as a central connecting point that links otherwise separate groups and supports information flow and collaboration.

**CoREN (Community Research and Engagement Network)**: A regional network established in 2019 to provide a sustained platform for the co-production of health and social care research, linking together communities, VCFSE organisations, practitioners and researchers.

**CoREN Leadership Group**: A collaborative group of nine members from commissioned VCFSE organisations who set the strategic direction of the CoREN and facilitate connections.

**Deliberative Democratic Theory**: A theoretical lens used in the evaluation that focuses on the quality of discussion and whether there is sufficient opportunity for inclusive participation. It judges legitimacy by interactions such as reason-giving, mutual listening, and the ability to challenge claims.

**Downstream impacts**: Unpredictable and diffuse later effects that occur after initial collaborations facilitated through the CoREN.

**Framework Analysis**: The qualitative data analysis method used in this evaluation, involving the application of a priori (pre-determined) and inductive (emerging) codes.

**HDRC (Health Determinants Research Collaboration)**: An external collaboration mentioned by a participant (specifically the Blackpool HDRC) as a comparative example of an organisation with clear lines of accountability.

**ICB (Integrated Care Board)**: NHS body responsible for planning and commissioning services at system level.

**Knowledge broker / brokering**: An intermediary role that connects people and information across groups and helps collaborations form.

**Knowledge Mobilisation**: Making knowledge usable and used, through activities that share, translate, adapt and apply research (often with practitioners, policy, communities), so it informs decisions, practice or further research.

**Logic Model**: A diagrammatic and structured description of how the inputs and activities of an initiative are expected to lead to outputs and outcomes.

**NIHR (National Institute for Health and Care Research)**: The UK funding body that funds the ARC NWC and positions public involvement as a core principle to ensure research reflects community needs.

**Partnership Synergy**: A theoretical concept referring to improved research outputs that result from organisations, communities, and individuals pooling their perspectives and resources rather than working alone.

**Public Involvement or Patient and public involvement and Engagement (PPIE)**: Terms that refer to patients and the public contributing to shaping and doing research, beyond being study participants. Research in this case is carried out ‘with’ or ‘by’ members of the public rather than ‘to’, ‘about’ or ‘for’ them.

**Projectisation**: A term used to describe a stop-start cycle of public involvement in research where engagement is limited to the lifespan of a specific grant, often reducing it to a temporary "box-ticking" exercise.

**Relational Approach (to Evaluation)**: An evaluation method that treats impact as the formation of new or reconfigured relationships, tracking subtle relational shifts that may enable later outcomes.

**REN (Research Engagement Network)**: An NHS England-funded initiative supporting VCFSE-led work to involve communities often excluded from research.

**Research literacy**: Knowledge and confidence that helps people understand research and take part in it on more equal terms.

**Social Capital**: The value that is generated from access to relationships and networks, such as trust and access to support.

**Stakeholder and Engagement Manager**: A full-time role funded by ARC NWC to support the CoREN, broker links between researchers and communities, and manage engagement.

**VCFSE (Voluntary, Community, Faith and Social Enterprise) Sector**: Umbrella term for charities, community groups, faith-based organisations and social enterprises. The CoREN aims to integrate this sector into research.
